# Monte Carlo characterization of biocompatible beta‐emitting ${}^{90}Y$ glass seed incorporated with the radionuclide ${}^{153}\text{Sm}$ as a SPECT marker for brachytherapy applications

**DOI:** 10.1120/jacmp.v14i5.4302

**Published:** 2013-09-06

**Authors:** Asghar Hadadi, Mahdi Sadeghi, Dariush Sardari, Alireza Khanchi, Alireza Shirazi

**Affiliations:** ^1^ Department of Medical Radiation Engineering Science and Research Branch Islamic Azad University Tehran Iran; ^2^ Agricultural, Medical and Industrial Research School Nuclear Science and Technology Research Institute Karaj Iran; ^3^ Nuclear Science and Technology Research Institute Tehran Iran; ^4^ Department of Biophysics Faculty of Medicine Medical Sciences University of Tehran Tehran Iran

**Keywords:** brachytherapy, dosimetry, YAS glass, TG‐60 protocol, Monte Carlo simulation

## Abstract

A glass seed consisting of the $\beta^{-}$‐emitting radionuclide ${}^{90}Y$ incorporated with radionuclide ${}^{153}\text{Sm}$ as SPECT marker is proposed for potential application in brachytherapy in order to reduce the undesirable dose to healthy adjacent organs. The aim of this work is to determine the dosimetric characteristics, as suggested in the AAPM TG‐60/TG‐149 reports, for this seed using Monte Carlo simulation. Monte Carlo codes MCNP5, EGSnrc, and FLUKA were used to calculate the absorbed dose distribution around the seed. Dosimetric parameters, such as reference absorbed dose rate, radial dose function, and one‐dimensional (1D) and two‐dimensional (2D) anisotropy functions, were obtained. The computational results from these three codes are in agreement within 5.4% difference on average. The absorbed dose rate at the reference point was estimated to be 5.01 cGy $h^{-1}\;\mu {\text{Ci}}^{-1}$ and self absorption of YAS glass seed amounted to 30.51%. The results showed that, with thermal neutron bombardment of 5 hours in a typical flux, sufficient activity for applications in brachytherapy may be achieved. With a 5 mCi initial activity, the total dose of a YAS glass seed was estimated to be 1.38 Gy at 1.0 cm from the seed center. Comparing with gamma emitting seeds, the ${}^{90}Y$ seed could reduce undesirable doses to adjacent organs, because of the rapid dose falloff of beta ray. Because of the high $R_{90}$ value of 5.5 mm, fewer number of ${}^{90}Y$ seeds will be required for an interstitial brachytherapy treatment using permanent implant, in comparison with other beta‐emitting seeds. The results would be helpful in the development of the radioactive implants using ${}^{90}Y$ glass seeds for the brachytherapy treatment.

PACS numbers: 87.53.Jw, 87.56.bg

## I. INTRODUCTION

Beta‐ray seeds offer several advantages over photon seeds. Beta particles have short ranges; therefore, they can give a potentially lower dose to adjacent critical organs. They are shielded easily, lowering extra dose to medical staff as well as the patient. Beta‐ray seeds such as ${}^{90}\text{Sr}/{}^{90}Y,{}^{90}Y,{}^{32}P,{}^{106}\text{Ru}/{}^{106}\text{Rh}$ are widely and routinely used for brachytherapy applications including intravascular brachytherapy (IVBT) and treatment of pterygia, choroid melanomas, and retinoblastomas.^(1)^

Several factors, such as the type and energy of radiation and half‐life, must be considered for radionuclide selection in permanent interstitial brachytherapy of selected localized tumors. Table 1 presents the comparison of the properties of several beta emitters.^(2)^ The radionuclide ${}^{90}Y$ is an ideal medical isotope with very interesting therapeutic features. This radionuclide is a pure beta emitter with high endpoint energy 2.284 MeV and half‐life 64.0 h. The maximum range of the ${}^{90}Y$ beta radiation in water is 11 mm, and 90% of the emitted energy is absorbed within a sphere of water with a radius of 5.3 mm.^(3)^ For the first time in 1956, Mallard et al.^(4)^ raised the idea of using the radionuclide ${}^{90}Y$ for treatment of pituitary tumors. Since then, ${}^{90}Y$ pituitary implants were used in the 1960s and 1970s as one of the first‐line treatments for pituitary tumors in many endocrine centers.^(5)^

In the field of cancer radiotherapy, rare earth aluminosilicate (REAS) glasses have been used as radioactive material vehicles because they can contain large amounts of beta‐emitting rare earth isotopes (e.g., ${}^{90}Y,{}^{166}\text{Ho},{}^{153}\text{Sm}$ and ${}^{165}\text{Dy}$ from 25 to 70 wt%). In this type of application, glasses should be biocompatible, nontoxic, and chemically insoluble, to prevent radioactivity leakage within the *in vivo* treatment site. REAS glasses are chemically durable and biologically inert *in* vivo.^(^ ^6^ ^,^ ^7^ ^)^ Another advantage of this glass is that the desired nonradioactive isotope is incorporated within the glass structure and activated *in situ* by neutron bombardment to form the beta‐emitting isotope and, hence, handling of radioactive materials during the manufacturing process is avoided.^(8)^ Yttrium aluminosilicate (YAS) glasses are of most interest among others because of their applicability in interstitial brachytherapy. YAS glasses, in the form of microspheres or seeds, have received attention in the treatments of liver cancer, rheumatoid arthritis, and prostate tumors.^(9)^ In 1987, Hyatt and Day^(8)^ studied ten YAS glasses, made by traditional high temperature melting, with yttria content varying from 25 to 55 wt%. They found that the VHN for YAS glasses is high compared to other glasses, so these glasses should display good resistance against abrasion. These glasses were also found to have very high chemical durability in distilled water. In 1993, Erbe and Day^(10)^ first reported that $17Y_{2}O_{3}‐19{\text{Al}}_{2}O_{3}‐64{\text{SiO}}_{2}\;(\text{mol}\;\%)$ (YAS) glass had most chemical durability in distilled water at 37°C and, based on their excellent chemical durability, YAS glass microspheres of 20–30 μm diameter were suitable for *in vivo* applications. Besides, In 1994, White and Day^(6)^ studied YAS glasses in the form of microspheres (20 to 40 μm in diameter) and seeds (with dimensions approximately 0.8 mm diameter and 5 mm length) to deliver large doses to sites inside the body.

Currently, bioactive and biodegradable glasses, made by sol‐gel technique, are also of interest for interstitial brachytherapy. Roberto et al.^(^ ^11^ ^,^ ^12^ ^)^ introduced the bioactive ${\text{SiO}}_{2}‐{\text{CaO}}^{‐152}{\text{Sm}}_{2}O_{3}$ glasses produced by the sol‐gel process for prostate cancer treatment. These glasses may be activated by neutron bombardment to form the beta‐emitting ${}^{153}\text{Sm}$ isotope. Preliminary studies for the implant of biodegradable radioactive ${}^{153}\text{Sm}$ seeds in the liver and brain have been previously published.^(^ ^13^ ^,^ ^14^ ^,^ ^15^ ^)^ Other investigators synthesized two sets of ceramic seeds by the sol‐gel technique with Si‐Ca‐Sm and Si‐Ca‐Ho incorporating natural samarium and holmium elements for use in brachytherapy.^(^ ^16^, ^17^, ^18^, ^19^ ^,^ ^17^ ^,^ ^18^ ^,^ ^19^ ^)^

**Table 1:** Properties of several beta‐emitting radionuclides

*Radionuclide*	*Half‐life*	*Maximum Energy (MeV)*	*Average Energy (MeV)*	*Abundance of Parent (%)*	*γ Emissions (keV)*	*Thermal Neutron Cross Section of Parent* (b)^a^
90Y	64.0 h	2.284 (100%)	0.934	100	Brems	1.28
90Sr	29.1 y	0.546 (100%)	0.196	‐	Brems	‐
$32_{P}$	14.3 d	1.710 (100%)	0.695	100	Brems	0.172
$186_{\text{Re}}$	90.6 h	1.076 (73.0%)	0.35	37.4	137 (8.65%)	112
${}^{188}\text{Re}$	17.0 h	2.119 (71.6%)	0.764	62.6	155 (14.9%)	76.4
${}^{153}\text{Sm}$	46.7 h	0.817 (21.0%)	0.228	26.75	103 (28.3%)	206
${}^{142}\text{Pr}$	19.1 h	2.159 (96.3%)	0.809	100	1580 (3.7%)	11.5
${}^{177}\text{Lu}$	6.71 d	0.497 (78.6%)	0.133	2.59	208 (11.0%)	2090
${}^{166}\text{Ho}$	26.8 h	1.856 (51.0%)	0.667	100	80.6 (6.2%)	64.7

a Mughabghab SF^(30)^

The updated American Association of Physicists in Medicine (AAPM) TG‐43U1 report recommended a dosimetry protocol for interstitial brachytherapy seeds.^(20)^ TG‐43U1 parameters are explicitly for low‐energy photon‐emitting brachytherapy seeds, such as ${}^{125}I$ and ${}^{103}\text{Pd}$, and beta‐emitting seeds were not included. The AAPM TG‐60/TG‐149 reports addressed intravascular brachytherapy physics and included the recommendation of dosimetry of beta emitters, such as ${}^{90}\text{Sr}/{}^{90}Y$ and ${}^{32}P$,^(^ ^21^ ^,^ ^22^ ^)^

The aim of the present work is to determine the dosimetric characteristics of $\beta^{-}$‐emitting YAS glass seed incorporated with ${}^{153}\text{Sm}$ as a SPECT marker for potential use in permanent or temporary interstitial implant for treating suitable tumors. The AAPM TG‐60/TG‐149 dosimetric parameters, such as reference absorbed dose rate, radial dose function, and one‐dimensional (1D) and two‐dimensional (2D) anisotropy functions, were calculated in water for a cylindrical seed using the Monte Carlo method based on the MCNP5, EGSnrc, and FLUKA radiation transport code systems. The dose rates in different radial distances from the seed and various angles, as well as isodose curves were derived. Moreover, the selfabsorption of the seed, $R_{90}$ value, and the activity of seed in thermal neutron flux for various bombardment times were evaluated. In order to investigate the viability of producing seed, the trial fabrication of the YAS glass seed with the isotope ${}^{152}\text{Sm}$ incorporated by sol‐gel technique was evaluated.

## II. MATERIALS AND METHODS

### A. Seed description

**Figure 1:**
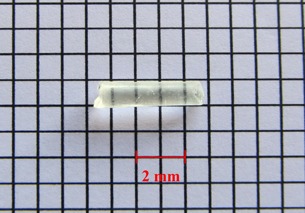
YAS glass seed incorporated with ${}^{152}\text{Sm}$ obtained by the sol‐gel process.

**Figure 2:**
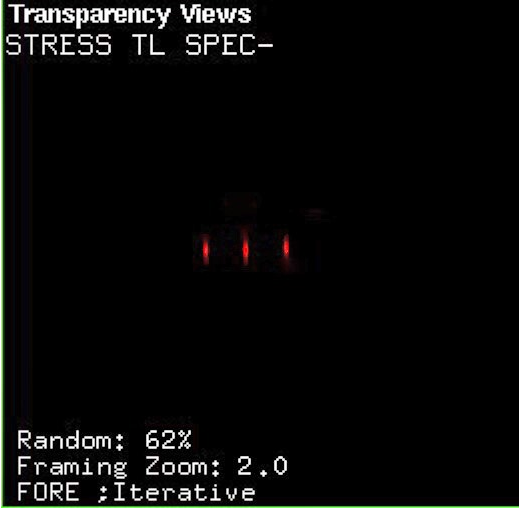
The SPECT image of three YAS glass seeds that were placed into the cylindrical water phantom.

The YAS glass seed is a cylinder 4.5 mm long and 0.8 mm diameter, the same as the typical ${}^{125}I$ seeds. The glass is composed of 43.2% Y, 7.9% Al, 14% Si, 34.7% O, and 0.2% ${}^{152}\text{Sm}$ (in wt %) with a density of $3.8\;g/{\text{cm}}^{3}$. The glass with composition of $Y_{2}O_{3}:\;55\%,\;{\text{Al}}_{2}O_{3}:\;15\%$, and ${\text{SiO}}_{2}:\;30\%$, that has been used in the work by Hyatt and Day,^(8)^ is transparent with a slight yellow tint. The used chemical precursors were TEOS $\left(\text{Si}({\text{OC}}_{2}{H_{5})}^{4},\;\text{Merck},\;98\%\right)$, yttrium nitrate hexahydrate $\left(Y({{\text{NO}}_{3})}^{3}.\;6H_{2}O,\;\text{Daejung},\;>\;99.9\%\right)$, aluminium nitrate nonahydrate $\left(\text{Al}({{\text{NO}}_{3})}_{3}.\;9H_{2}O,\;\text{Merck},\;>\;98.5\%\right)$, and enriched samarium oxide $\left({}^{152}{\text{Sm}}_{2}O_{3},\;\text{ISOTEC},\;98.7\%\;\right)$. The deionized water and ethanol (Carlo Erba, 99.8%) as a solvent, nitric acid (2N) as an acid catalyst, and formamide (${\text{HCONH}}_{2},\;\text{Merck},\;\geq \;99.0\%$) as a modifier, were used in the sol–gel preparation. All reagents were of analytic grade. The samarium was introduced during the sol–gel synthesis as a solution prepared by solubilization of the oxide with nitric acid. After mixing the above composites and homogenization (stirred for 30 minutes on a magnetic agitator heated at 60°C), the mixture was placed in Teflon molds, with cylindrical punctures, so that they could acquire the desired seed format. When the solution appeared like a gel, it was dried in an electric oven at 110°C, about 20 hours. Then, the dried seeds were removed from the molds and put in a ceramic crucible and subjected to heat treatments carried out at 500°C for 1 hour and at 1000°C for 24 hours. Because the thermal treatment is carried out at high temperature, the glass‐forming elements are closely bound together; therefore, after implantation, they will not be released into the body. However, these elements are not harmful to the body. In order to assess the leakage of seed, the active ${}^{90}Y$ glass seed was kept inside the distilled water, then the water was analyzed for a period of two weeks. After this period, there was no evidence of leakage in water. Figure 1 illustrates the sample of desired glass seed. Because of high density of YAS glass, a radio‐opaque marker is not necessary; however, the 103 keV gamma ray emitted by ${}^{153}\text{Sm}$ can be detected by a gamma camera.^(23)^ Also, cladding or encapsulation is not required because the seed is nonreactive in water or tissue. The YAS glass seed may be activated by neutron bombardment in a thermal neutron flux density typical of research reactors to form the ${}^{90}Y$ and ${}^{153}\text{Sm}$ radionuclides. Table 2 presents several radio‐nuclides that are made during activation of YAS glass seed.^(2)^ The activity of seed in thermal neutron flux of $3\;\times \;10^{13}\;{\text{cm}}^{-2}s^{-1}$ was calculated using FLUKA code. The activities of ${}^{90}Y,{}^{31}\text{Si},{}^{28}\text{Al}$, and ${}^{153}\text{Sm}$ isotopes and total activity of seed were calculated to be 5.750, 0.208, 11.894, 0. 95, and 17.852 mCi after 5 hours irradiation, respectively. Due to low thermal neutron cross section, the production of ${}^{90m}Y$ and ${}^{19}O$ were ignored. With a cooling time of 13 hours after the irradiation end (approximately five times the half‐life of ${}^{31}\text{Si}$), the activities of ${}^{31}\text{Si}$ and ${}^{28}\text{Al}$ isotopes are not considered to be significant. At this time, the activity of ${}^{90}Y$ isotope is approximately 5 mCi, equivalent to values used in brachytherapy, and the activity of ${}^{153}\text{Sm}$ isotope is approximately 0.8 mCi, enough to be suitable for SPECT imaging. Three YAS glass seeds containing ${}^{152}\text{Sm}$ were irradiated for 30 hours in the 5 MW research reactor TRR in an irradiation position, with thermal and epithermal neutron fluxes of $2.3\;\times \;1012\;n.{\text{cm}}^{-2}s^{-1}$ and $2.5\;\times \;1010\;n.{\text{cm}}^{-2}s^{-1}$, respectively. After 5 hours cooling time, these seeds were placed with 1 cm distance from each other into the cylindrical water phantom with a diameter of 15 cm and a length of 20 cm. Figure 2 illustrates the SPECT image of these seeds that was obtained using DST‐XL dual‐head gamma camera system. It should be noted that with increase in thermal neutron flux density or yttrium and samarium content in the seed, the required irradiation time for desired activity is decreased. Also, the seed could be used for an HDR treatment if a high neutron flux reactor having more than $1015\;{\text{cm}}^{-2}s^{-1}$ becomes available.

**Table 2:** Radionuclides from neutron activation of YAS glass

*Radionuclide*	*Thermal Neutron Cross Section of Parent (b)*	*Half‐life*	*Radiation*	*Yield*
${}^{90}Y$	1.28^a^	64.1 h	β 2.284 MeV	100%
${}^{90m}Y$	0.001^b^	3.19 h	γ 480 keV	90.0%
${}^{153}\text{Sm}$	206.00^a^	46.7 h	β 0.817 MeV γ 103 keV	21.0% 28.3%
${}^{31}\text{Si}$	0.107^a^	2.62 h	β 1.492 MeV γ 1.27 MeV	99.9% 0.07%
${}^{28}\text{Al}$	0.231^a^	2.24 m	β 2.864 MeV γ 1.78 MeV	100% 100%
${}^{19}O$	0.00016^a^	26.9 s	β 4.819 MeV γ 1.36 MeV	56.1% 50.3%

a Mughabghab SF^(30)^

b Handbook on nuclear activation cross‐sections^(31)^

### B. Dose calculation formalism

For deriving dosimetric parameters, the dose calculation formalism for beta seeds recommended by the AAPM TG‐60/TG‐149 reports was adopted in this work. For a beta‐emitting seed, the reports recommend, for reference absorbed dose rate in water ${}_{w}(r_{0},\;\Theta_{0})$, the reference point should be $r_{0}\;=\;2\;\text{mm}$ and $\Theta_{0}\;=\;90{}^{\circ}$ on the transverse axis. A line‐source approximation, with the same effective length of the seed, for geometry function $G_{L}(r,\Theta )$ was assumed when it was used to derive radial dose function ${}_{\text{gL}}(r)$ and the anisotropy function F(r,Θ).

### C. Monte Carlo simulation

An important advantage of Monte Carlo (MC) simulations is that the dose distribution can be calculated with high spatial precision. Due to the extremely large dose gradient around the beta‐ray sources, it is difficult to obtain accurate dose distribution experimentally. Therefore, MC simulation is widely used as an alternative means to determine the dose distribution around the radioactive seeds for therapeutic purposes. A variety of well‐validated general‐purpose MC codes are currently available for brachytherapy dosimetry. In this study, the MCNP5 (version 1.5.1) Los Alamos National Laboratory, Los Alamos, NM), EGSnrc (version 4 2.3.2) (NRC, Ottawa, Canada), and FLUKA (version 2011.2.8) (www.fluka.org ) Monte Carlo codes were used to calculate the quantitative dosimetric parameters of the ${}^{90}Y$ seed.

MCNP is a general‐purpose, time‐dependent Monte Carlo transport code. It can be used for neutron, photon, electron, or coupled neutron/photon/electron transport. It can model an arbitrary three‐dimensional geometry and various source types such as point, surface, and volume with user‐defined source spectrum. The photon energy regime is from 1 keV to 100 GeV, and the electron energy regime is from 1 KeV to 1 GeV. The electron physics in MCNP is essentially that of the ITS3.0 and uses the Goudsmit‐Saunderson multiple scattering theory. The user creates an input file that is subsequently read by MCNP. The user can instruct MCNP to make various tallies related to particle current, particle flux, and energy deposition. A pulse height tally F8 provides the energy distribution of pulses created in a detector by radiation.^(24)^

The EGSnrc system of computer codes is a general purpose package for the Monte Carlo simulation of the coupled transport of electrons and photons in an arbitrary geometry. The dynamic range of charged particle kinetic energies begins from a few tens of keV up to a few hundred GeV, and dynamic range of photon energies lies between 1 keV and several hundred GeV. This code includes a variety of general purpose user RZ codes for cylindrical geometry problems such as DOSRZnrc, FLURZnrc, CAVRZnrc, and SPRRZnrc. The user code DOSRZnrc calculates absorbed dose in any cylindrical geometry. The RZ codes systematically make use of 15 different types of source geometries including parallel beam source, point source, circular source, and cylindrical source. The EGSnrc code adopts the new multiple scattering model PRESTA‐II that has been developed by Kawrakow et al.,^(25)^ which makes a significant advance in the science of electron transport.

FLUKA is a multipurpose Monte Carlo code which can simulate with high accuracy the interaction and propagation in matter of about 60 different particles. The dynamic ranges of photon and electron energies go from 1 keV to thousands of TeV. Moreover, time evolution and tracking of emitted radiation from unstable residual nuclei can be performed online by this code. Decay scoring is one of the valuable features of this code that has been used in this study. FLUKA can handle even very complex geometries, using an improved version of the well‐known Combinatorial Geometry (CG) package. The various source types such as point, spherical shell, cylindrical shell, Cartesian shell, and spherical surface can be defined in this code. The electron transport in FLUKA is based on its code EMF. This code adopts Ferrari‐Sala multiple scattering model, which is essentially based on Moliere's theory.^(26)^

**Figure 3:**
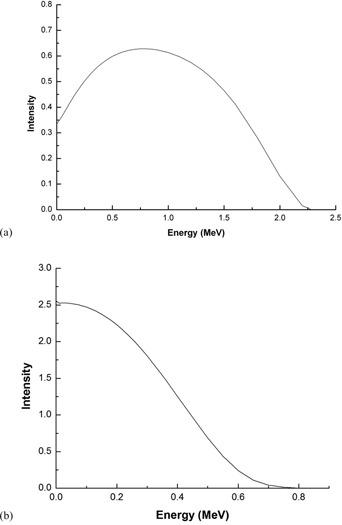
The beta‐ray spectrum of: (a) ${}^{90}Y$, (b) ${}^{153}\text{Sm}$.

The input parameters commonly used such as the materials and geometry of source and the cutoff energies for electron and photon were exactly the same for these codes. A cylindrical volume source, with composition that is shown in Table 3, was modeled. The radionuclide was assumed to be uniformly distributed in the source. The beta spectrum used for the MC simulations is provided in Fig. 3.^(2)^ The dosimetric data in the scoring zones were calculated at radial distances from the source from 1 to 10 mm in 0.5 mm increments, and over angles ranging from 0° to 90° in 10° increments.

MCNP5 simulations were carried out with the center of the cylindrical source placed at the center of a 20 cm radius spherical water phantom, approximating a semi‐infinite water phantom, allowing for full electron scattering conditions in the region of interest. The scoring geometry is composed of an array of ring shaped volumes. These volumes are defined by the intersection of a series of concentric spherical shells with a series of concentric cones, both originating at the center of the cylindrical source. The thicknesses of spherical shells are 0.1 mm, and the angular aperture difference of two adjacent concentric cones defining the cell is 2°. The *F8 tally was used to score the energy deposited in the scoring cells around the source. Dose was calculated with *F8 tally divided by mass of scoring cells. Mode P E was used with default modeling of bremsstrahlung. MCPLIB04 and el03 cross‐sectional libraries were used for the electron and photon‐coupled transport. The cutoff energies for electron and photon were taken as CUT:E = 0.04 MeV and CUT:P = 0.01 MeV, and the number of electron substeps per energy step, ESTEP value, was set as built‐in default adjusted for all materials. Moreover, an ITS‐style energy‐indexing algorithm was used for a more accurate sampling energy straggling. This algorithm serves to reduce the frequent repetition of unwanted imposition of linear interpolation on partial steps, and to allow more balance among excursions above and below the energy groups from which the Landau sampling was made.^(24)^

**Table 3:** Composition of YAS glass seed

*Element*	*Weight (%)*	*Mass in Seed (mg)*
Y	43.2	3.722
O	34.7	2.994
Si	14.0	1.203
Al	7.9	0.679
${}^{152}\text{Sm}$	0.2	0.02
Total	100	8.618

EGSnrc and FLUKA simulations were carried out with the center of the cylindrical source placed at the center of a cylindrical water phantom with a radius of 20 cm and a length of 40 cm. DOSRZnrc user code, in case of EGSnrc, and USRBIN card in case of FLUKA, were used to score the dose in the scoring zones around the source. A set of thin, short cylindrical shell segments, concentric about the long axis of source, was adopted as scoring zones. In our calculations, the radial thickness of the cylindrical shell segments was taken as 0.1 mm and their length was 0.2 mm. The cutoff energies for electron and photon were identical to that of the MCNP5 code and were taken as AE = ECUT = 0.551 MeV and AP = PCUT = 0.01 MeV for both EGSnrc and FLUKA codes. The maximum fractional energy loss in an electron step, ESTEPE value for EGSnrc, and WHAT (2) in EMFFIX card for FLUKA, was set at 0.07. In FLUKA simulations, instead of using beta spectrum that is shown in Fig. 3, the SDUM parameter in BEAM card was taken as ISOTOPE and option HI‐PROPErt was set for ${}^{90}Y$ and ${}^{153}\text{Sm}$ radionuclides.

Monte Carlo simulations were carried out on a personal computer with an Intel Core i7–3.5 GHz CPU (Intel Corporation, Santa Clara, CA) and Windows 7 (Microsoft, Redmond, WA) Ultimate OS for MCNP5 and EGSnrc codes, and Fedora 15 (Red Hat; Raleigh, NC) linux‐based OS for FLUKA code. The number of source electron histories was set at $2\;\times \;10^{8}$ for each simulation in order to obtain a statistical error less than 1% at the reference point.

## III. RESULTS & DISCUSSION

### A. The radial dose profile and reference dose rate

**Figure 4:**
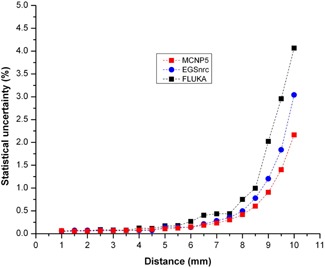
Relative statistical uncertainties of MCNP5, EGSnrc, and FLUKA codes as a function of distance on the transverse axis.

**Figure 5:**
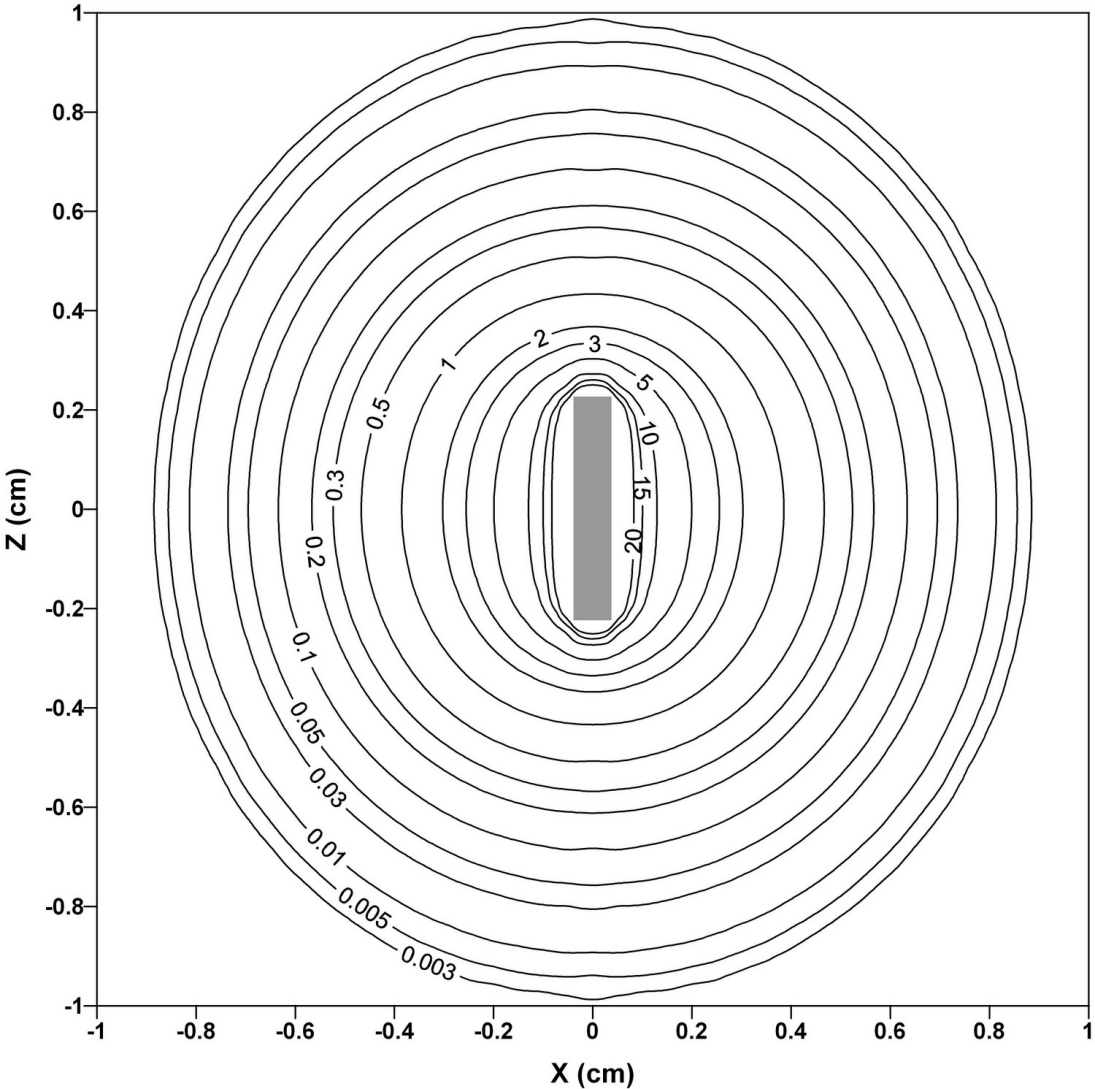
Isodose contour map around the ${}^{90}Y$ glass seed. The labels of the isodose lines are in units of cGy/h/μCi.

Table 4 presents the radial dose rate values from MCNP5, EGSnrc, and FLUKA simulations, at different distances from center of the seed on the transverse axis. As shown in this table, the results of the three codes are in good agreement with each other. With $2\;\times \;10^{8}$ histories, the relative statistical uncertainties at reference point were 0.06%, 0.07%, and 0.06% for MCNP5, EGSnrc, and FLUKA codes, respectively. Figure 4 illustrates the statistical uncertainties for these codes as a function of distance on the transverse axis. These uncertainties correspond to one standard deviation. As can be seen from Fig. 4, statistical uncertainties are less than 1% to 8.5 mm, and MCNP5 code has statistical uncertainties less than two other codes. The reference absorbed dose rates per unit activity for glass seed that are calculated using the above mentioned codes, are presented in Table 5. The average of depicted values, $5.01\;\text{cGy}\;h^{-1}\;\mu {\text{Ci}}^{-1}$, was chosen as the reference value for glass seed. The reference value for this glass seed is higher than the values of 3.30, and $2.412\;\text{cGy}\;h^{-1}\;\mu {\text{Ci}}^{-1}$, calculated for Novoste Beta‐Cath ${}^{90}\text{Sr}/{}^{90}Y$ seeds, and ${}^{142}\text{Pr}$ glass seed, respectively.^(^ ^22^ ^,^ ^7^ ^)^ The uncertainties in this table are associated with relative statistical uncertainties of Monte Carlo simulation. The two‐dimensional dose profiles from MCNP code simulation are observed in Fig. 5. In this figure, the interpolation was performed using the kriging algorithm in the SURFER program (Golden Software Inc., Golden, CO).

**Table 4:** Monte Carlo‐calculated radial dose rate of ${}^{90}Y$ glass seed

*Radial Distance (mm)*	*Dose Rate (cGy/h/μCi)*		
	*MCNP5*	*EGSnrc*	*FLUKA*
1.0	15.1808	14.8252	15.1650
1.5	8.3660	8.1798	8.3662
2.0	5.0542	4.9524	5.0244
2.5	3.2021	3.1369	3.1440
3.0	2.0809	2.0366	2.0120
3.5	1.3742	1.3373	1.2952
4.0	0.9000	0.8799	0.8346
4.5	0.5858	0.5750	0.5326
5.0	0.3784	0.3706	0.3375
5.5	0.2383	0.2331	0.2081
6.0	0.1445	0.1428	0.1256
6.5	0.0844	0.0837	0.0727
7.0	0.0470	0.0468	0.0400
7.5	0.0246	0.0246	0.0211
8.0	0.0121	0.0121	0.0104
8.5	0.0054	0.0055	0.0047
9.0	0.0022	0.0022	0.0019
9.5	0.0008	0.0008	0.0008
10.0	0.0003	0.0003	0.0003

**Table 5:** The absorbed dose rates at the reference point in water

*Monte Carlo Code*	${Ḋ}_{W}(r_{0},\theta_{0})\;(\text{cGy}/h/\mu \text{Ci})$
MCNP5	5.0542±0.0029
EGSnrc	4.9524±0.0035
FLUKA	5.0244±0.0030
Average	5.0103±0.0018

### B. Radial dose function $g_{L}(r)$

The radial dose function, $g_{L}(r)$, was defined in order to characterize the effects of absorption and scatter in the medium along the transverse axis of the seed. The values of radial dose function are calculated using MCNP5, EGSnrc, and FLUKA codes and are presented in Table 6. The $g_{L}(r)$ values from these codes are in good agreement with each other; the agreement between MCNP5 with EGSnrc is especially significant. A fifth‐order polynomial fit of the MCNP5 radial dose function can be expressed as:

(1) $$g_{L}(r)=a_{0}+a_{1}r+a_{2}r^{2}+a_{3}r^{3}+a_{4}r^{4}+a_{5}r^{5}$$

where $a_{0}\;=\;1.02772,a_{1}\;=\;0.18833,a_{2}\;=\;‐0.13205,a_{3}\;=\;0.0172,a_{4}\;=\;‐7.31\;\times \;10^{-4}$, and $a_{5}\;=\;4.06\;\times \;10^{-6}$, and define correlation coefficient R = 0.99998.

**Figure 6:**
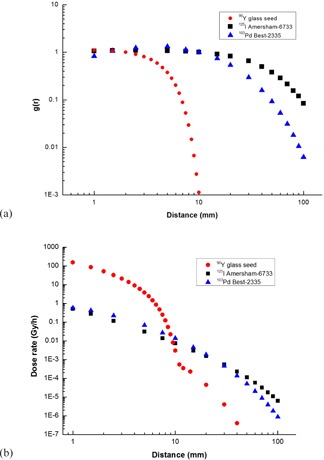
Comparison of the Monte Carlo‐calculated radial dose function (a) and initial dose rate (b) of the ${}^{90}Y$ glass seed with those of the ${}^{125}I$ and ${}^{103}\text{Pd}$ seeds.

**Figure 7:**
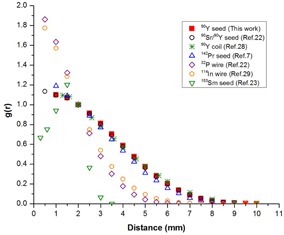
Comparison of the Monte Carlo calculated radial dose function of the ${}^{90}Y$ glass seed with those of other betaemitter seeds.

Figure 6(a) shows a comparison of radial dose functions of ${}^{90}Y$ glass seed, Amersham Health model 6733 ${}^{125}I$ seed,^(27)^ and Best Medical model 2335 ${}^{103}\text{Pd}$ seed.^(27)^ As can be seen in this logarithmical figure, the radial dose function profiles for ${}^{125}I$ and ${}^{103}\text{Pd}$ seeds are approximately flat to 10 mm, while that of ${}^{90}Y$ glass seed falls off to 0.1% at the same distance. The comparison of initial dose rate of these seeds is provided in Fig. 6(b). The air‐kerma strengths, $S_{K}$, for ${}^{125}I$ and ${}^{103}\text{Pd}$ seeds, which commonly used for prostate implants, are selected to be 0.75 U and 2 U, respectively. Also, the initial activity of ${}^{90}Y$ seed is 1 mCi. As shown in this figure, the initial dose rate adjacent to the ${}^{90}Y$ seed is much higher than those of ${}^{125}I$ and ${}^{103}\text{Pd}$ seeds, but decreases sharply to 41% of that of ${}^{125}I$ seed at 10 mm. After 11 mm, the maximum range of ${}^{90}Y$ beta radiation, the initial dose rate of ${}^{90}Y$ seed is due to bremsstrahlung radiation and is significantly lower than those of ${}^{125}I$ and ${}^{103}\text{Pd}$ seeds. At 40 mm, the initial dose rate of ${}^{90}Y$ seed decreases to approximately 0.3% and 0.2% those of ${}^{103}\text{Pd}$ and ${}^{125}I$ seeds, respectively, and therefore the absorbed dose by the healthy adjacent organs is decreased. However, it should be noted that when the effective distance of seed is so short, the seed's ability to deliver effective dose to the whole irregular shaped tumor may be limited. In Fig. 7, the radial dose function of the ${}^{90}Y$ glass seed is compared to those of the several beta‐emitter seeds.^(^ ^7^ ^,^ ^22^ ^,^ ^23^ ^,^ ^28^ ^,^ ^29^ ^)^ As observed in figure, the radial dose function of this seed is approximately similar to those of the ${}^{90}\text{Sr}/{}^{90}Y$, and ${}^{142}\text{Pr}$ seeds. Also, the decrease in radial dose function for that is slower than to those of the ${}^{153}\text{Sm},{}^{32}P$, and ${}^{114}\text{In}$ sources. The falloff rates are related to the beta energies, and therefore ${}^{90}Y$ with the highest mean beta energy has the slowest falloff.

**Table 6:** Monte Carlo‐calculated radial dose function $g_{L}(r)$ for the ${}^{90}Y$ glass seed

*Radial Distance (mm)*	$g_{L}(r)$		
	*MCNP5*	*EGSnrc*	*FLUKA*
1.0	1.100	1.100	1.108
1.5	1.070	1.070	1.076
2.0	1.000	1.000	1.000
2.5	0.912	0.917	0.905
3.0	0.810	0.816	0.792
3.5	0.703	0.697	0.664
4.0	0.587	0.586	0.548
4.5	0.475	0.475	0.433
5.0	0.374	0.371	0.332
5.5	0.282	0.285	0.251
6.0	0.202	0.201	0.174
6.5	0.137	0.138	0.118
7.0	0.088	0.089	0.075
7.5	0.053	0.055	0.046
8.0	0.030	0.031	0.026
8.5	0.015	0.015	0.013
9.0	0.007	0.007	0.006
9.5	0.003	0.003	0.003
10.0	0.001	0.001	0.001

### C. 1D and 2D anisotropy functions

The 2D anisotropy function F(r,θ) accounts for the variation in dose distribution around the seed as a function of polar angle relative to the transverse plane. The 1D anisotropy function, $\varphi_{\text{an}}(r)$, is identical to the anisotropy factor defined by the original TG‐43 protocol.^(20)^ The values of the 1D and 2D anisotropy functions that are calculated using MCNP5 code are listed in Table 7. The maximum value of the 2D anisotropy function was calculated to be 6.785.

### D. The self‐absorption of the seed and $R_{90}$ value

With a 604.0 h half‐life, 90% of the dose from ${}^{90}Y$ isotope is deposited in 8.86 days. Due to interseed attenuation, the part of beta energy is deposited within the seed. The self‐absorption of ${}^{90}Y$ glass seed was calculated to be 30.25% and 30.77% using EGSnrc and FLUKA codes, respectively. Therefore, the contained activity of this seed is approximately 1.4 times the apparent activity. The $R_{90}$ value is defined as the radius of a sphere around the seed in water in which 90% of the output energy of seed is absorbed. The $R_{90}$ value for ${}^{90}Y$ glass seed, which was calculated using FLUKA simulation, was found to be 5.5 mm, which is in agreement with that found by Dezarn et al.^(3)^

## IV. CONCLUSIONS

${}^{90}Y$ is a pure beta‐emitting isotope with interesting therapeutically features for potential use in cancer treatment. In this study, a β‐emitting YAS glass seed incorporated with ${}^{153}\text{Sm}$ as a SPECT marker, with dimensions of 0.8 mm diameter and 4.5 mm length, is proposed for application in permanent interstitial implantation of selected localized tumors. This seed is nonreactive in the biological tissue; therefore, encapsulation is not required. The MCNP5 (version 1.5.1), EGSnrc (version 4 2.3.2), and FLUKA (version 2011.2.8) Monte Carlo codes were used to calculate the quantitative dosimetric parameters, suggested in the AAPM TG‐60/TG‐149 reports. The input parameters used were exactly the same for each code, to ensure that the differences in calculated results are only due to the differences in physical modeling between these codes. The results of these codes are in good agreement with each other. The YAS glass seed has desirable dosimetric properties. The dose rate in the reference point was calculated to be $5.01\;\text{cGy}\;h^{-1}\;\mu {\text{Ci}}^{-1}$, and average of self absorption of YAS glass seed was estimated to 30.51%. The $R_{90}$ value was found to be 5.5 mm, equivalent to volume of approximately $0.7\;{\text{cm}}^{3}$. Because of the high $R_{90}$ value of ${}^{90}Y$ seed, a fewer number of seeds will be required for an interstitial brachytherapy treatment using permanent implant in comparison with other beta‐emitting seeds. With 5 hours for irradiation time in thermal neutron flux of $3\;\times \;10^{13}\;{\text{cm}}^{-2}s^{-1}$ and 13 hours for cooling time, the seed activity of approximately 5 mCi may be achieve. With this initial activity, the total dose of YAS glass seed was estimated to 1.38 Gy at 1.0 cm from the seed center on the transverse axis. As shown in Fig. 2, the 103 keV gamma ray emitted from glass seed incorporated with ${}^{153}\text{Sm}$ can be detected by a gamma camera. It should be noted that the YAS glass seed is reusable after a HDR afterloading brachytherapy route by reactivation. Also, unused seeds are reusable by reactivation. It should be considered that because of relatively short half‐life of ${}^{90}Y$, the YAS glass seed generally has a high initial dose rate. For such a high dose rate seed, an implant other than the precise seed placement could significantly under‐ or overdose the target. Moreover, the process of seed production using sol‐gel method is time‐consuming, and due to shrinkage and deformation during the sol‐gel process, the seed manufacturing is relatively difficult.

**Table 7:** Monte Carlo‐calculated anisotropy function F(r,θ) for the ${}^{90}Y$ glass seed

	*r (mm)*																		
Θ *(deg)*	*1.0*	7.5	*2.0*	*2.5*	*3.0*	*3.5*	*4.0*	*4.5*	*5.0*	*5.5*	*6.0*	*6.5*	*7.0*	*7.5*	*8.0*	*8.5*	*9.0*	*9.5*	*10.0*
0				0.856	0.939	0.979	0.955	1.036	1.094	1.162	1.318	1.352	1.645	1.967	2.317	2.774	3.860	5.544	5.886
10				0.948	0.944	0.966	0.998	1.048	1.102	1.189	1.292	1.422	1.623	1.893	2.313	2.901	3.841	5.146	6.785
20		1.110	1.133	1.007	0.989	1.005	1.033	1.079	1.132	1.205	1.302	1.430	1.607	1.854	2.186	2.728	3.557	4.789	5.982
30	1.051	1.064	1.085	1.048	1.034	1.042	1.066	1.099	1.141	1.202	1.285	1.398	1.545	1.750	2.052	2.505	3.169	4.068	4.906
40	1.021	1.045	1.063	1.055	1.048	1.055	1.071	1.099	1.132	1.180	1.250	1.334	1.455	1.616	1.810	2.117	2.668	3.313	3.914
50	1.011	1.029	1.043	1.043	1.042	1.050	1.061	1.084	1.104	1.145	1.186	1.250	1.328	1.445	1.591	1.805	2.101	2.498	2.790
60	1.006	1.016	1.025	1.027	1.029	1.034	1.041	1.055	1.068	1.093	1.115	1.152	1.196	1.267	1.356	1.484	1.619	1.912	1.920
70	1.003	1.008	1.012	1.011	1.014	1.017	1.021	1.027	1.033	1.043	1.055	1.077	1.096	1.124	1.146	1.225	1.283	1.354	1.411
80	1.001	1.002	1.004	1.003	1.004	1.003	1.005	1.009	1.007	1.010	1.015	1.025	1.025	1.034	1.039	1.056	1.060	1.090	0.949
90	1.000	1.000	1.000	1.000	1.000	1.000	1.000	1.000	1.000	1.000	1.000	1.000	1.000	1.000	1.000	1.000	1.000	1.000	1.000
$\varphi_{\text{an}}(r)$	1.107	1.345	1.313	1.406	1.277	1.208	1.178	1.167	1.165	1.179	1.205	1.251	1.310	1.399	1.522	1.708	1.987	2.374	2.668

## ACKNOWLEDGMENTS

The authors are thankful to the Isotopes Research Group for allowing the accomplishment of this work in the scope of their professional and academic activities.
